# Leveraging digital health systems maturity assessments to guide strategic priorities

**DOI:** 10.4102/jphia.v15i1.769

**Published:** 2024-12-09

**Authors:** Phiona Vumbugwa, Nancy Puttkammer, Moira Majaha, Andrew Likaka, Sonora Stampfly, Paul Biondich, Jennifer E. Shivers, Kendi Mburu, Olusegun O. Soge, Chris Longenecker, Jan Flowers, Caryl Feldacker

**Affiliations:** 1 Department of Global Health, University of Washington, Seattle, United States of America; 2 Department of Global Health, International Training and Education Centre for Health/University of Washington, Seattle, United States of America; 3 The Biomedical Research and Training Institute, Harare, Zimbabwe; 4 Directorate of Quality Management and Digital Health, Ministry of Health, Lilongwe, Zambia; 5 Global Health Informatics, Regenstrief Institute, Indiana, United States of America; 6 Program for Appropriate Technology in Health, Washington DC, United States of America; 7 School of Medicine, University of Washington, Seattle, United States of America; 8 Department of Laboratory Medicine and Pathology, University of Washington, Seattle, United States of America; 9 Division of Cardiology, University of Washington, Seattle, United States of America

**Keywords:** digital health, health information systems (HIS), digital health systems maturity assessments (DHSMA), health systems strengthening (HSS), digital health governance, eHealth

## Abstract

**Background:**

Many low- and middle-income countries (LMICs) face the daunting task of digitising, maturing and deciding where to invest in digital health systems.

**Aim:**

Describing the facilitators and barriers to conducting digital health maturity assessments and how health leaders can prioritise the assessments.

**Setting:**

eHealth leaders from 10 African countries, working or supporting Ministries of Health’s digital health and participating in the eHealth Leaders’ Forum from July 2023 to September 2023.

**Methods:**

This qualitative, descriptive study utilised key informant interviews conducted via Zoom with 14 conveniently selected leaders. We used Dedoose Version 9.0 to develop themes based on the health system’s building blocks.

**Results:**

Participants identified maturity assessments as a critical first step to digital health strengthening, showing the system’s performance and building a baseline response to systematic data quality challenges. Barriers to conducting digital health maturity assessment include lacking collaborators’ buy-in, fragmented vision, overdependence on donor priorities, non-supportive policies and an inadequately skilled workforce. Facilitators include multi-stakeholder engagement, understanding the country’s digital health ecosystem and appropriately integrating maturity assessment objectives. Recommendations include capacity building in data use and conducting maturity assessments at all health system levels to grow the demand and value of digital health strengthening.

**Conclusion:**

Promoting digital health maturity assessments can help leaders to make appropriate decisions to prioritise areas of improvement and steward maturity advancement as a pathway to strengthening the health system.

**Contribution:**

We spotlight the perspectives of African eHealth leaders, centering voices on the barriers, facilitators to planning and recommendations for implementing digital health systems maturity assessments.

## Introduction

Health information systems (HIS) are critical for health systems strengthening (HSS). Global health organisations endeavour to create digitised and integrated HIS with the capacity to collect, collate and analyse vast amounts of information for rapid response to public health needs.^1,2^ A digitised health system obtains, effectively uses, and securely exchanges information to improve public health practice and population health outcomes.^3^ Digital health systems provide fast, reliable and efficient ways for governments to track public health interventions but establishing and maintaining them can be costly.

The coronavirus disease 2019 (COVID-19) pandemic exposed gaps in how digital health supports information sharing for decision-making.^4,5^ Many low- and middle-income countries (LMICs) have developed national digital health strategies utilising the World Health Organization (WHO)-International Telecommunications Union (ITU) National eHealth Strategy Toolkit, with strategic objectives for creating a more governed and managed health system.^6,7^ The WHO-ITU National eHealth Strategy toolkit is a framework and method for developing a national eHealth vision, action plan and monitoring framework.^8^ Post-COVID-19, several digital systems implemented for early detection, reporting and response across government systems and partners are yet to be evaluated in the light of guidance to integrate the systems.^9^ The lack of interoperable information systems and limited implementation of automatic data exchange threatens health systems’ functioning and performance. Strengthening a digitised health system requires understanding strengths, gaps and maturity. This points to the need for an appropriate framework to track and assess a system’s core functions and capabilities.^10,11^

Digital health systems maturity assessments (DHSMA) are often conducted as part of a governance function to learn and provide evidence for strategic investment in HIS. We define maturity as the degree to which a digitised HIS is interoperable, scalable, offers security and privacy, complies with healthcare standard regulations, and makes health information readily available.^4,5^ In contrast to health systems assessments that may focus across health system building blocks, DHSMA focuses on reviewing an organisation’s processes, capabilities and performance against either internal or external standards, with specific reference to the essential components that make up a well-functioning HIS.^12^ Digital health systems maturity assessments allow an organisation to benchmark itself over time and lay out a pathway for strategic improvement over time. Assessing digital health systems’ maturity is important to know what has been tried and/or done to scale or where strategic objectives still fall short. Health leaders need proven tools to assess the maturity of their digital health systems.

Conducting maturity assessments supports the implementation of the WHO-ITU National eHealth Strategy Toolkit. A few countries in Africa, such as Kenya, Ethiopia, Ghana and Zambia, have conducted maturity assessments; however, these have not been conducted consistently.^13,14^ Repeating HIS maturity assessments over time allows for longitudinal monitoring of HIS maturity and provides the follow-up to verify that recommendations were implemented.^14^ There is little published literature on the utility of HIS maturity assessments to health system leaders and planners and the facilitators and barriers to conducting HIS maturity assessments in LMICs are not described.

**FIGURE 1:**
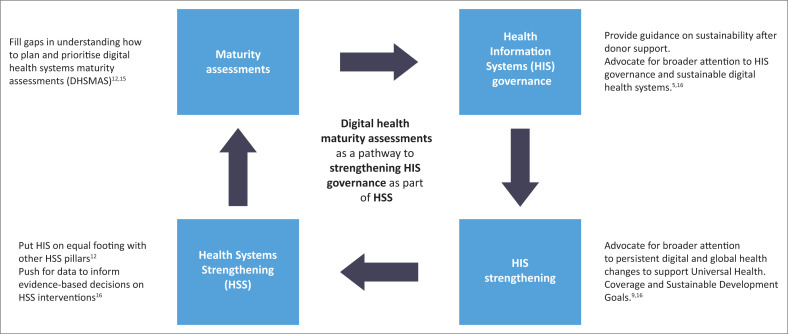
Conceptual framework for digital health systems maturity assessments and the contribution to health systems strengthening.

Our study explores (1) health leaders’ perceptions of the value and importance of digital health maturity assessments as part of HIS governance and strategic planning, (2) barriers and facilitators to planning a maturity assessment and (3) health leaders’ recommendations for overcoming barriers to conducting digital health maturity assessments. The study provides information relevant for the institutionalisation of HIS maturity assessments as a critical ingredient for health system governance and holistic HSS as represented in Figure 1.

## Research methods and design

### Study design

The project used a qualitative descriptive design to assess health leaders’ perceptions of barriers, facilitators and recommended strategies for conducting maturity assessments. Key informant interviews were conducted using a structured key informant guide.

### Study setting and participants

The study was conducted with African health directors from LMICs participating or supporting a global health informatics leaders’ network. In 2023, I-TECH Digital Initiatives Group (DIGI), in partnership with Regenstrief Institute, launched the eHealth Leaders Forum community of practice (eHLF CoP) for national health information leaders in the Ministry of Health (MoH). The eHLF provides peer learning, networking and a place to share best practices. Health leaders discuss digital health implementation, share challenges faced and opportunities for resources or research and offer peer support in digital/HISs assessments, planning and improvement. Through monthly forum meetings, health leaders expressed the need for support in analysing and selecting interventions that strengthen digital health. As champions of digital health in respective countries, the setting provided a platform to interact with skilled and knowledgeable participants on the subject matter.

eHealth Leaders Forum is one of several initiatives for digital health capacity building supported by the United States (US) Centers for Disease Control and Prevention’s (CDC) Technical Assistance Platform (TAP). eHealth Leaders Forum is part of the overall TAP capacity development strategies, including digital health training for senior or mid-level leaders and using informatics-savvy health organisation (ISHO) assessment tools at national and sub-national levels.^15^ All MoH respondents participated in eHLF and some but not all the respondents were exposed to other TAP capacity development interventions.

### Participant selection, recruitment and eligibility

Health leaders were selected using a convenience sample from 10 countries. Participants either had a leadership position in the MoH at the director level (*n* = 10) or represented partner organisations funding or supporting digital health innovations in the countries (*n* = 4). The leaders participated in or supported the eHLF. To manage selection bias and generalisability, participants were stratified to ensure representation from different regions of Africa. To enhance the validity of results, participants were asked to share examples to support their responses. Although the representation sample size was small (10 African leaders), the key results were validated during the eHLF meeting where more than 20 other health leaders were present.

All participants had at least 2 years of experience in their roles and were expected to be conversant with health informatics systems or the digital health ecosystem. Participants with less than a year of experience in their roles and those who did not respond to a second follow-up email were excluded.

An introductory message was developed and initial contact with countries’ MoH digital health leaders was made through email and share the study information. The study team used the email to follow-up and to recruit participants. All participants recruited for the study had been engaged in eHLF for at least 6 months. At least one health leader per organisation and country was purposively selected to participate.

### Data collection

The key informant guide questions were structured and aligned with the six building blocks: governance and leadership, health information, health workforce, financing, medicines and technologies and service delivery^16^ (see Appendix 1). All participants received an initial email message seeking consent to participate in the study. A discussion format was used in a 30 min virtual interview (via Zoom) scheduled at the participant’s preferred time to solicit and probe more on responses, with participants providing supporting documents where applicable. The interviews were recorded and in cases where the discussion exceeded 30 min, permission to proceed was sought from the participant. Participants answered questions based on their knowledge and shared strategic documents supporting their responses. Interview saturation was reached once participants responded to questions with the same ideas as in earlier responses.

### Data analysis

The interview transcripts were analysed using Dedoose Version 9.0. We developed a codebook and coding linked to the interview questions. Inductive and deductive themes emerged as we analysed the codes for each transcript. Inter-coder reliability was performed with primary and secondary coders by defining the codes, testing coding together, independent coding and discussion after coding. Reliability was tested using Cohen’s kappa formula and coders’ agreement 0.81 of the coding decision. Qualitative thematic analysis was conducted to identify themes relevant to each specific pillar, while content analysis was used to summarise evidence of best practices to support narratives.^17,18^ Themes for contribution to digital health maturity assessments matched best practices drawn from supporting documents. Recommendations on prioritising and conducting digital health maturity assessment were assigned priority levels from highest to lowest based on the frequency with which the theme was mentioned across respondents.

### Ethical considerations

Ethical clearance to conduct this study was obtained from the University of Washington (UW) internal review board (IRB) Human Subjects Division (HSD) reviewed and approved the research under the UW IRB STUDY00018156.

All participants provided formal verbal consent prior to an interview and consent to record the discussion, which was manually transcribed. We confirm that all necessary participant consent has been obtained and the appropriate institutional forms have been archived, and that any patient, participant, or sample identifiers included were not known to anyone (e.g., hospital staff, patients or participants themselves) outside the research group so cannot be used to identify individuals.

To ensure anonymity, participants were allocated numbers and recordings were started after the demographic data were collected. No study documentation or information was shared via WhatsApp to maintain confidentiality. All personally identifiable information was anonymised during transcription, and WhatsApp was encrypted to ensure data privacy; transcripts were stored on SharePoint in a password-protected computer.

## Results

A total of 14 interviews were conducted; 12 were males, while 2 were females (Table 1). All participants occupied the deputy director level or above. Of the 14 participants, 10 were national level representatives from MoH, universities, and implementing partners (labelled as ‘MoH’) and 4 were representatives from funding and global organisations (labelled as ‘PO’). Among all participants, six had more than 8 years; four had 5 to 7 years; and 4 had 2 years to 4 years of working experience leading digital health in their respective organisations. All five regions of the African continent were represented. We provide the participant number, age, and organization (01/44/MoH) for each participant quote.

**TABLE 1:** Participants’ profile.

Institution	Total	National level	International level
Ministry of Health	7	7	0
Universities	2	2	0
Implementing partners	1	1	0
Funding partner	2	0	2
Global organisation	2	0	2

**Total**	**14**	**10**	**4**

In the following sections, we report findings under the three objectives.

### Objective 1: Digital health maturity assessment contribution to strengthening health systems

Digital health systems maturity assessments were identified as a critical beginning step to strengthening health systems. Several countries had not conducted DHSMA. Health leaders cited maturity assessments contributions including systems performance review, prioritisation of resources, needs and strategies.

All participants agreed that there is a huge gap in conducting DHSMAs in their countries as a national approach, with only 3 out of 10 countries attesting to having conducted a digital health maturity assessment in the past 3–5 years. Similarly, one participant stated that strategic planning for DHSMAs has neither been performed nor prioritised at the continent level. Some participants confirmed being involved in implementing DHSMAs as a once-off, research-based or nationwide activity. Respondents cited that most of these initiatives were donor-driven; hence, they lacked follow-up to recommendations and ownership of results and did not prioritise countries’ specific needs.

Participants indicated that a DHSMA contributes to knowing the system’s performance and understanding the gaps and strengths to build a baseline to strengthen digital health. Other participants acknowledged that maturity assessments provide a platform to respond to systematic challenges and recommendations on data quality seen through data quality assurance (DQAs) and monitoring and evaluation (M&Es), which most organisations support.

### Summary of objective 1

Table 2 summarises the contribution of digital health maturity assessments to strengthening HIS and provides examples of corresponding best practices from evidence cited by participants.

**TABLE 2:** Summary of digital health maturity assessments contribution to health information systems strengthening.

Summary point	Quote	Best practice cited by key informants
Identifies focus areas that need to be addressed in a digital health system, its strengths and prioritise action.	‘If you don’t know the existing systems’ capacity and maturity, how can you make decisions and allocate resources to support this? So, the maturity assessment is critical in providing leaders with the information they need to plan and support efficient and optimal digital health strengthening.’ (Participant 05, 39 years, MOH)	Zambia conducted the ISHO maturity assessments to strengthen the performance of the SmartCare digital health system.
Provides data for evidence-based decisions, including policies and guidelines that prioritise interventions to strengthen the digital health system.	‘After we did the assessment, where we found the most weaknesses is where we focused. But I’m not necessarily saying that the other areas were not focused on. Still, we were able to focus on that area and have a number of initiatives listed in this area of workforce get to the level that we would want to be as a country.’ (Participant 08, 36 years, MOH)	Kenya developed an online human resource capacitation platform, training on digital health systems as part of yearly accreditation.
Provides better advocacy opportunities for political will and prioritisation of resource allocation towards interventions to strengthen a digital health system.	‘… [*W*]e have that’s what we call ANICiiS, which is the entity, but before that had to do all kinds of assessment in the health sector to see what the gaps are, what needs to be done, which resulted in what we call the digital plan for the country.’ (Participant 12, 42 years, MOH)	The Democratic Republic of Congo’s national Digital Health System budget is 80% funded by the government’s post-digital health maturity assessments.
Tracks longitudinal evolution and progress of digital health system performance and in providing accurate data	‘… [*A*]fter the previous assessment, we then realized there are a number of issues that we needed to add looking at infrastructure, governance, the establishment of a health informatics and data analytics department, which is one critical area and addresses some issues pertaining to governance.’ (Participant 03, 46 years, MOH)	Ethiopia conducted an ISHO assessment in 2020 and followed up with another assessment in 2023 to track the progress of its digital health system roadmap.
Digital health maturity assessments provide an opportunity to work on systematic challenges seen in DQAs and M&E, which most programme budget allocations prioritise	‘Currently, we are planning another level of assessment because there’s some level of inconsistency with HIV data in Nigeria. So, we didn’t want to determine the exact number of people who are currently on treatment because our spectrum data was showing that Nigeria has just reached maturity level on treatment, yet we are still having new patients on treatment.’ (Participant 01, 44 years, MOH)	Nigeria is planning an ISHO assessment to validate the performance of the digital health systems.

DQA, data quality assurance; M&E, monitoring and evaluation; ISHO, informatics-savvy health organisation; MoH, Ministry of Health; KIIs, key informants.

### Objective 2: Facilitators and barriers to digital health maturity assessments

#### Governance and leadership

Government support and political will were identified as the main facilitators for promoting digital health maturity assessment. Policies and best practices backed by the MoH’s digital health system priorities were considered as effective motivators for implementation. Policies reportedly empower health leaders by creating an enabling environment and allowing resource allocation to build a strong base for assessing digital health:

‘At the national level, a policy could create a national health information exchange between agencies, public and private, and would help to improve the coordination of tracking implementation through digital health maturity assessment.’ (Participant 06, 45 years, PO)

Participants cited the lack of collaborators’ buy-in and shared vision as barriers to digital health maturity assessments. Strategic plans are not enough to encourage engagement in digital health assessments without partner support. One respondent stated that:

‘Once we have that five-year strategic plan, we pick out key flagship activities, and we got this digitalization and the strengthening of the health information systems assessments. Once we do that, we engage with the member states ministries to get their buy-in, but you will not probably get 100% of the support, which is like one weakness or challenge because we don’t impose ourselves.’ (Participant 11, 43 years, MoH)

Another respondent echoed the lack of support from the government and policymakers and its impact on digital health assessments and strengthening:

‘In some countries, there is a lack of shared vision to invest in digital health. This can make it difficult to get the resources needed to plan maturity assessment and sustain digital health systems strengthening.’ (Participant 02, 45 years, MoH)

#### Health financing

The availability of dedicated funding for digital health systems was seen as a conduit to facilitate digital health maturity assessments. Participants acknowledged the advocacy surrounding the importance of digital health and its benefits, with some governments beginning to fund digital health systems interventions and infrastructure. Three participants from national and international levels gave an example of best practice in the Democratic Republic of Congo, where the government finances 80% of digital HISs, with 1.5% of the national health budget invested towards strengthening digital health systems.

All participants firmly acknowledged donor support, including the development of district health initiatives and electronic health records (EHR) systems, as important to start conversations around maturity assessment and assessing scalability. One participant stated:

‘The openness by the government to receive financing from donors for the assessments, that’s an opportunity …’ (Participant 01, 44 years, MoH)

All participants acknowledged that financing for digital health remains the most significant challenge impacting efforts to prioritise planning of digital health systems assessments and overall system strengthening. It was commonly reported that a specific national HIS allocated budget was lacking. One key informant stated:

‘Looking at advocacy around M&E has been shown partly by having these critical positions in place but is not complimented by a budget to say if we have at least 8%–10% of the ministry budget committed to M&E and informatics, then we know that the oil of the system is guaranteed. So, we find that advocacy is not there in terms of its translation into tangible activities due to poor funding. So, I would advocate that 8%–10% of the ministry budget always support the HIS’s M&E, including assessments, health informatics, and systems strengthening.’ (Participant 09, 52 years, PO)

Most participants agreed that over-reliance on donors and funders prevents flexibility in planning for digital health assessments, which are generally not planned as part of the restricted funding. The same is seen to have placed over-dependency on donor priorities with less prioritisation or room to negotiate for financing other critical competing priorities such as maturity assessments. In support, a few participants indicated that their country’s budget to roll out the District Health Information System (DHIS2) is funded by the Global Fund and the President’s Emergency Plan for AIDS Relief (PEPFAR). One respondent stated:

‘In terms of specific budget to support, like DHIS2 hardware and infrastructure, that has been donor funded. Much of the support for DHIS2, if not mistaken, about 80% comes from the Global Fund, 20% is from PEPFAR, and 0% is from the government. Right now, we are rolling out EHR, electronic medical record; much of the support is coming from PEPFAR followed by Global Fund, and assessing their maturity is not part of the grant.’ (Participant 04, 51 years, MoH)

Several participants indicated that having outside funders as the leading financial supporters severely impacts the ability of a country to prioritise and promote interventions that support digital health maturity assessments, as donors dictate the priority of the funding. One participant agreed, saying:

‘… [*W*]hat is not going well is that the financing structure has been too donor oriented, so the priorities have been donor orientated.’ (Participant 12, 42 years, MoH)

#### Workforce

Participants noticed the lack of training and skillset in maturity assessments for health workers who are essential health data collectors and users as a barrier to collecting valuable and credible data for prioritising digital health assessment planning. One participant supported saying:

‘… [*S*]kills and training of health workers in maturity assessment are limited, and that is a barrier, as it means that they may not be able to provide the most up-to-date information to show the performance of the system; there is a delay in getting this data to make decisions, delaying planning on appropriate digital health interventions.’ (Participant 11, 43 years, MoH)

Skilled informatics and maturity assessment workforce were cited as vital in successfully implementing digital health assessments, yet countries lack personnel who can lead or conduct digital health assessments. Most health directors cited a lack of recognition for health informatics roles within the health workforce and a lack of power to negotiate priorities to focus on digital health being a barrier to digital health systems assessments. The lack of motivation and poor working conditions, including long hours, low pay and inadequate resources, contributed to governments’ inability to retain key informatics skilled staff.

It was commonly observed that countries lack specific training structures for health informatics or digital health personnel, as digitisation of HIS has only recently become part of the health system structure. Two participants from national universities echoed that training has often been ad hoc and developing standardised training programmes is challenging and happening slowly:

‘At the moment, the highest level of training that we have in the country for health information people is a diploma. We don’t yet have a degree that particularly looks at Health Information System, so that’s the first one.’ (Participant 10, 46 years, PO)

Respondents believed that improving these conditions could motivate leaders to invest in the digital health workforce, which is key in digital health assessments and overall healthcare improvement.

Most participants, 11 of 14, echoed that the current workforce structure facilitates the capacity of available health workers with knowledge of the importance of conducting digital health maturity assessments. Participants cited methods such as including digital health systems mentorship or training in license renewal platforms, departmental mentorships to review reports, data use, and tailored training on digital health for specific needs. One participant stated:

‘… [*D*]igital health systems training should start from where the health providers like the nurses, pharmaceutical, laboratory technologists are trained at the colleges, universities, and so on, They can be introduced to health information systems, management of health information systems so they can start having a good understanding about information systems, the benefits, uses, challenges so that when they go out there, the workforce can be already empowered.’ (Participant 05, 51 years, MoH)

All the above were seen as facilitators to appropriately integrate maturity assessment objectives as the workforce understands the country’s digital health ecosystem and priorities better, thus making planning easy. Across the board, all respondents noted that the workforce’s skill set is essential in ensuring a well-functioning digital health, with the ability to plan and conduct assessments effectively and implement any required changes noted.

#### Infrastructure and medical supplies

Most participants believed the diversity of systems, the heterogeneity in investments in information communication technology (ICT) infrastructure, and digital health supplies to be significant barriers for many LMICs to plan for digital health maturity assessments due to cost and availability. Also, the expensive technology has led to having several fragmented digital health systems, either disease-specific or programme-specific and non-interoperable; as such, integrating digital health maturity assessment plans becomes a challenge. Many cited this as why digital health is not prioritised over other health system pillars. The digital health infrastructure should be defined to plan for a maturity assessment, as explained by one participant:

‘I think the infrastructure is a real challenge because we need to ensure that we have connectivity, which is one of the challenges, apart from the equipment like servers, firewalls, and switches that are too expensive to buy and maintain, thus makes digital health systems assessment less priority, we only replace the piece not functional.’ (Participant 08, 36 years, MoH)

A few participants brought up an important point that for the few countries that have made steady progress in planning and implementing digital health assessments, the biggest challenge has been having standard and/or user-friendly tools, standardising and having interoperable systems as countries use different electronic medical records (EMR) systems, such as open-source medical records system (OpenMRS), laboratory and management information systems (LAMIS) and others. Many participants stated that each implementing partner would have its own unique system, resulting in a disintegrated digital health landscape, making it challenging to prioritise their maturity assessment planning as systems need to be separated because their maturity and implementation may not be comparable:

‘So, there are multiple systems that are in use and coming, and they are not interoperable. As a result, there is duplication and redundancy in that aspect; we find it hard to pick which one to strengthen.’ (Participant 13, 51 years, PO)

Uniquely, one participant pointed out that infrastructure for HISs falls under different departments and is regulated by ministries, like the Ministry of ICT or Finance. This makes it challenging for the MoH to prioritise digital health systems funding and planning digital health maturity assessments as the infrastructure belongs to a different ministry as cited by one participant:

‘Secondly, in many ministries of health, infrastructure is not necessarily the Ministry of Health’s concern, but it is maintained by another ministry, for example the Ministry of ICT, which also get funds from the Ministry of Finance. The fact that the infrastructure is domiciled in another ministry is also a challenge for the health ministry.’ (Participant 02, 45 years, MoH)

Stating something almost similar, several respondents cited technology evolving quickly and infrastructure becoming outdated or incompatible faster than strengthening processes can catch up, posing a challenge for keeping digital health assessment planning and implementation up to speed.

### Summary of objective 2

A summary of barriers and facilitators to planning digital health maturity assessments is provided in Table 3.

**TABLE 3:** Summary of barriers and facilitators to planning digital health maturity assessments.

HSS pillar	Barriers	Facilitators	Examples of digital health strategic documents
Leadership and governance	Lack of collaborators’ buy-in, shared vision and supporting policies.	Coordinated implementation of digital health policies through a national HIS ministry	Ministry of Public Health, DRC. National Development Plan Health Informatics 2020–2024.^19^
Financing	There is no specific national digital health system budget to support digital health maturity assessments.	Allocate a specific national digital/HIS strengthening budget	Democratic Republic of The Congo Ministry of Public Health Quantified Roadmap of Digital Health Investments.^20^
Information systems	Fragmented systems that rely on power and connectivity.	Open-Source Systems that are interoperable and standardised building from external collaborations	Ministry of Public Health, DRC. National Development Plan Health Informatics 2020–2024.^19^
Workforce	Inadequate technically capacitated staff to properly understand, use, maintain and manage digital health systems infrastructure and technology.	Curriculum for digital health systems workforce and health workers orientation	Kenya’s Virtual Academy online training for health workers.^21^ Zimbabwe and Ethiopia informatics training within the University of Zimbabwe and the University of Gondar
Service delivery	Competing priorities Siloed and fragmented digital health systems divorced from the national HIS structure make choosing or integrating systems to focus maturity assessment difficult.	Collaborating with other organisations and agencies, leveraging partnerships to provide resources, expertise and support for digital health strengthening efforts	DRC’s One Health digital health system Zambia’s SmartCare System
Medical supplies	A lack of standard/user-friendly tools costly equipment and infrastructure, including maintaining server rooms, providing reliable connectivity at all levels.	Flexible resource allocation systems	Integration of mobile technology to supplement HIS

HIS, health information systems; HSS, health systems strengthening.

### Objective 3: Ways health leaders can better plan for digital health maturity assessments

Participants provided various ways health leaders can better plan digital health maturity assessments in their countries.

All participants recommended multi-stakeholder collaborative engagement when planning maturity assessments, from the idea’s conception to completion. Several participants attested that engagement is crucial to ensure that all parties understand and agree on the objectives and methodology of the assessment, leverage existing M&E systems and piggyback on already established systems, such as the DHIS2 monitoring, to effectively plan for a digital health maturity assessment. One participant emphasised that:

‘First of all, the methodology must be understood in a consultative process supported by widespread stakeholder engagement early on in the process including both public and private sector within the health information area. The second part will be after that widespread consultative process, I would want the objectives for the National Health Information System maturity assessment to be outlined and linked to the strategic plan of the digital health system for all implementers’ involvement.’ (Participant 10, 46 years, PO)

In support of the aforesaid, several participants agreed that for digital health maturity assessment planning to be effective, there needs to be strong collaborative engagement of all stakeholders to advocate for government prioritisation and political will to support the initiative.

Several participants stated that collaborators should be involved in planning a maturity assessment based on their context. Another participant opined that stakeholders hold different powers and expertise, which is key when planning a maturity assessment for the country. Participants outlined the need to bring a sense of recognition, ownership and support to plan a participatory action-based maturity assessment. More than half of the participants supported planning for action by prioritising using context-based assessment tools, minimising duplication of activities, and letting countries decide the processes. In support, one participant echoed that:

‘Stakeholders should not be restricted only to MoH’s HIS and digital health department but include all partners, implementing, funding, Ministry of Information and the regulatory bodies, telecommunications [*public and private*], power supply organizations, community representatives and advocates. Including such key stakeholders necessitates them to understand the need for prioritizing digital health assessments and their role in setting goals and ownership of recommendations.’ (Participant 14, 41 years, MoH)

Almost all (*n* = 11/14) participants highly prioritised increasing the use of data when planning for digital health maturity assessment. Respondents indicated that the available systems’ data should be used to show the system’s weaknesses or strengths for the leaders to focus the assessment. A few also cited that stakeholders should have access to and the ability to analyse or report the data, which would prepare them to understand assessment findings and take ownership of improving the system.

### Summary of objective 3

Table 4 summarises recurring recommendations from participants.

**TABLE 4:** Summary of recommendations of ways health leaders can better plan for digital health systems maturity assessments.

Recommendations when planning a maturity assessment	Key quote	Priority
Collaborative engagement through stakeholder mapping	‘A multi-stakeholder, a multi-organization, multidisciplinary approach to the assessment, so ensuring that there’s a lot of good participation from a key set of diverse stakeholders …’ (Participant 06, 45 years, PO)	High
Identify the country’s priorities and choose appropriate implementation tools, implementers, time and process.	‘And finally, relevance to the local context, so, for the assessment to work well, it should be relevant to that local context so that findings apply to those specific needs of each country or organization. The data collection tool itself should be flexible to meet those needs.’ (Participant 07, 45 years, PO)	High
Increase utilisation of available health data to grow the demand and value of digital health and have its assessment prioritised.	‘I think we need to be very specific in the way that we solve the problems by using tools, for instance, these same digital health maturity tools, break them down even to the lowest level and keep ourselves alive all the time, like the way that people report monthly, or weekly, to keep health information systems alive or used through reporting and assessments …’ (Participant 13, 51 years, PO)	High
Capacity building at all levels to ensure scalability and continuity of the assessment process	‘One is that country leaders need capacity building and more sensitization. Some of these health leaders don’t understand the digital health terminology, and the leaders need to be sensitized to the health information systems used to give more support.’ (Participant 03, 46 years, MoH)	High
Identify DQA and M&E planned and include a digital health assessment to provide a cost-effective approach to conducting assessments that can be implemented at routine intervals.	‘… [*A*]lso, being flexible in the data collection and in the assessment tool itself and applying scalability to already existing plans like M&E and DQA conducted by partners can be cost-effective and promote continuity of digital health maturity assessment, to address different types of systems sizes and complexities and having a cost-effective approach to conducting assessments …’ (Participant 11, 43 years, MoH)	Medium
Advocate for increased political will, government ownership, inclusion in strategic plans and investment into digital health maturity assessments	‘I feel like the best recommendation I would have is having the political will for and understanding the need for accurate data, then it is easy to mobilize for resources to ensure that maturity assessments are planned and happening because there is some form of accountability.’ (Participant 12, 42 years, MoH)	Low

DQA, data quality assurance; M&E, monitoring and evaluation.

## Discussion

Health leaders recognised the value of having evidence from DHSMAs to guide them in planning for HIS strengthening. They recognised that DHSMAs provide a critical first step to enhance a system’s performance status, highlighting areas to integrate, expand and scale up towards the long-term goal of a functional, optimised, sustained and strengthened health system. Barriers to implementing maturity assessments include a lack of skilled workforce knowledgeable in digital health maturity assessments, fragmented digital health systems using expensive infrastructure and a lack of financing. Key facilitators to implementing digital health maturity assessment included coordination, collaborating with existing M&E programmes and knowledgeable health workers to conduct digital health assessments at all health facilities. Addressing these barriers and facilitators is crucial for effective digital health strengthening and data-driven decision-making in healthcare systems.

Governance is critical to HSS; maturity assessments, especially participatory assessments, can help strengthen that. Political will in LMICs can be a stepping stone to developing digital health policies that strengthen DHSMA to not only rely on international aid. Weak health systems governance in LMICs has resulted in fragmented or ad hoc health policy formulation, poising challenges in implementing DHSMA and impacting efforts to strengthen overall health systems.^22,23^ Leadership and governance for digital health include having multisectoral digital health steering committees such as an eHealth Technical Working group that oversees the implementation of digital health, interoperability activities and financial resourcing to aid the implementation of recommendations from the assessment.^14,24,25^ Information derived from maturity assessments can benefit digital health governance in: (1) lobby for political will to finance digital health systems, (2) providing guidance for improvement in health systems’ policies and (3) improving efficiency, effectiveness, performance and productivity in the whole health system.^26^ Through participatory planning, health systems governance leadership in Ghana and Rwanda effectively prioritised areas to improve digital health, supported by strong political will and governance structures.^27,28^ The LMICs governments should start growing focus on making the DHSMAs sustainable for a health system to function optimally.

Growing knowledge about the value of a digital health maturity assessment is critical to their sustainable implementation. The over-reliance on donor funding has led to digital systems being heavily supported by international organisations in many LMICs, affecting long-term implementation and sustainability of digital health systems, as objectives shift to those of funders. Firstly, making DHSMAs routine and operationalised as part of a strategic vision could increase demand for sustainable digital health assessments. Secondly, efforts should focus on building the culture of conducting regular DHSMAs using integrated and decentralised approaches. Integration supports participatory planning to co-develop the goals/objectives of the assessment based on country needs, priorities and collaborators’ implementation efforts.^29^ Participatory planning addresses not only technical aspects but also cultural, structural and governance-related factors that contribute to an effective maturity assessment. Thirdly, adapting existing digital health assessment tools such as ISHO and Stages of Continuous Improvement (SOCI) to the country context could aid in routinising DHSMAs without high costs.^16,30,31^ Fourthly, strengthening the capacity of health leadership in planning and conducting systems performance monitoring at all health facilities promotes accountability to health data, increasing data use. When the health team understands their responsibility, accountability and teamwork are cultivated, essential to improving data use, quality for informed decision-making, policy change and planning.^5,25^ All these factors could potentially sustain implementation and maturity assessments using available resources without relying much on donor funds.

Although there is limited literature on barriers and facilitators to implementing DHSMAs, our findings resonate to some recommendations where maturity assessments have been conducted outside Africa. Provider experiences, usability of data collection tools, and systems interoperability were challenges cited in conducting maturity assessment in Australia, resonating with our findings.^32^ In the US and Asia, context-based maturity assessments coupled with provider knowledge were key drivers to successful and sustainable assessments for their patient monitoring systems.^33,34^ Our study’s themes about routinising DHSMAs align with the US Public Health Informatics Institute’s concept of using assessments for advancing US county-level public health departments as ‘informatics-savvy organisations’.^12^ Countries realise the benefits and value of conducting digital health maturity assessments when governments implement the recommendations into binding policies and HSS activities.

Digital health maturity assessments are critical to establishing an evidence base and process for systematically prioritising objectives in the health sector. Digital health systems maturity assessments have the potential to focus resources, de-duplicate work, reduce staff workload and strengthening health systems. Because the digital health landscape and context will evolve over time, assessments should not be conducted as a one-time marker but as part of a routine iterative cycle. Also, longitudinal countrywide maturity assessments should be conducted for understanding digital health progress, updating the strategic vision, strategic objectives and country policies. Health leaders must have a shared vision and skilled champions to plan and/or implement the activities, financing and coordination for sustainable solutions that do not rely on international aid alone.^35^ This approach can be a pathway to ensuring the DHSMA results will be relevant and useful to all critical partners supporting digital health systems beyond donor-driven investments and projects.

### Limitations

Firstly, most countries had not conducted a maturity assessment at the time of interviews, so participant knowledge was based on M&E or demographic health surveys, which did not focus on digital health systems. Secondly, most (80%) key informants were health leaders from Africa who held positions by appointment and were engaged through eHLF, so their responses may have been biased and not have represented all health system leaders in LMICs. However, we expect the barriers, facilitators and recommendations they named would resonate with other LMIC regions as their responses were based on their area of expertise. Thirdly, the research did not ask about the drawbacks of conducting maturity assessments or why they might not bring value to HIS, which may have introduced bias by conveying an assumption that digital health maturity assessments are important. Lastly, the structure of questions resulted in confounding responses, with some participants treating HIS strengthening and digital health assessments interchangeably. All responses were clarified to ensure the correct meaning in interpretation.

## Conclusion

Strengthening HISs is vital in improving healthcare for all in LMICs. With the growing access to technology and increasing demand for digital health solutions, assessing their maturity to aid in identifying digital health priorities plays a vital role in improving HSS. Countries still face challenges in conducting digital health maturity assessments and operationalising results to strengthen their HIS. The challenges include lack of prioritisation of digital health because of low political will, a lack of shared vision as a result the donor-dependent funding of digital health system and a lack of essential skills in the health workforce to conduct maturity assessments. Addressing these barriers is crucial for planning for and executing digital health maturity assessments, potentially achieving effective HIS strengthening through data-driven decision-making in healthcare systems. Key to planning an effective digital health maturity assessment includes multi-collaborative engagements, contextualising to country needs and priorities, using existing resources and structures or M&E plans, advocating for government prioritisation and gaining political will. Institutionalising digital health maturity assessments as part of digital health governance offers a promise to adopt and build a foundation for having interoperable, integrated and sustainable HIS integral to a well-functioning and strengthened health system. There is a need for further longitudinal country-led DHSMAs, including a systematic study of their impact in improving the planning of digital health strategies and coordinating of investments. Government ownership of DHSMAs and related digital health initiatives will strengthen the policy environment for successful digital information systems in LMICs.

## Appendix 1

### Key informant guide

Today’s interview focuses on digital health information systems (DHIS) maturity. When we talk about DHIS maturity, please think about your national HIS ecosystem, rather than a specific paper-based or electronic data system. Please think comprehensively about the people, process and technologies that make up the DHIS to be mature, their role in prioritisation and optimising a strengthened system.

1 Please share a brief overview of your role within your organisation and years of experience.
2 What have been your country’s recent experiences with strategic planning for advancing the maturity of your national DHIS?
  - What processes or stakeholders would your organisation prioritise when planning systems’ capacity improvement?

### Governance and leadership

3 What policies are available for leader to motivate evidence-based action planning for DHIS?
  - Are there any best practices that you can call out from your country?
  - How do these policies empower leaders to promote HIS strengthening at organisational, national, regional or primary level?
  - What changes would you suggest to governance and leadership to prioritise DHSMA?

### Health information system

4 What are the motivators for prioritising DHIS assessments?
  - Describe any challenges.
  - Any suggestions to improve DHSMA and strengthening?

### Health financing

5 Tell me about financing for HIS strengthening, what is going on well, what are the gaps?
  - What opportunities are available in health financing that can motivate DHSMA?

### Health workforce

6 How do current health workforce systems/structure motivate DHIS maturity assessments?
  - What are the constraints to getting maturity assessments conducted?
  - What changes are needed in health workforce systems/structures, in order to advance the progress of DHIS workforce strengthening?

### Service delivery

7 What does a good DHIS maturity assessment system and service look like/cover to you?
  - What do you think are the important aspects for having a strengthened DHIS?

### Medical supplies

8 DHIS relies on commodity availability; what challenges do health leaders face in prioritising the commodities or infrastructure for DHIS?
  - What are the recommendations for prioritising DHIS supplies to strengthen health systems?

### Overall

9 To wrap up, what are your 2–3 main messages on the recommendations to better plan and prioritise DHIS maturity assessments to support systems strengthening?
  - Is there anything else you would like to add or comment on strengthening HSS in general?

## References

[1] ITU. Digital health platform handbook: Building a digital information infrastructure (infostructure) for health [homepage on the Internet]. [cited 2024 Feb 15]. Available from: https://www.itu.int:443/en/publications/ITU-D/Pages/publications.aspx

[2] Global strategy on digital health 2020–2025. Geneva: World Health Organization; 2021 [cited 2024 Feb 15]. Available from: https://www.who.int/docs/default-source/documents/gs4dhdaa2a9f352b0445bafbc79ca799dce4d.pdf

[3] McNabb SJN, Shaikh AT, Haley CJ. Modernizing global health security to prevent, detect, and respond. London: Elsevier, 2023; 572 p.

[4] Transform Health. Closing the digital divide: More and better funding for the digital transformation of health. [homepage on the Internet]. 2022 [cited 2024 Feb 15]. Available from: https://transformhealthcoalition.org/wp-content/uploads/2022/10/Closing-the-digital-divide-mainReport.pdf

[5] Mirza M, Grant-Greene Y, Valles MPJS, et al. Leveraging PEPFAR-supported health information systems for COVID-19 pandemic response. Emerg Infect Dis. 2022;28(Suppl 1):S49–S58. 10.3201/eid2813.220751 36502426 PMC9745247

[6] Weltgesundheitsorganisation, Internationale Fernmelde-Union, editors. National eHealth strategy toolkit. Geneva: World Health Organization; 2012.

[7] Mahama PN-j, Kabo-bah AT, Falchetta G, et al. Leaving no disease behind: The roadmap to securing universal health security and what this means for the surveillance of infectious diseases in Ghana as a precedent for sub-Saharan Africa. PLoS One. 2023;18(4):e0284931. 10.1371/journal.pone.0284931 37093834 PMC10124850

[8] McGuire F, Revill P, Twea P, Mohan S, Manthalu G, Smith PC. Allocating resources to support universal health coverage: Development of a geographical funding formula in Malawi. BMJ Glob Health. 2020;5(9):e002763. 10.1136/bmjgh-2020-002763 PMC7493092 32938613

[9] Ibeneme S, Karamagi H, Muneene D, Goswami K, Chisaka N, Okeibunor J. Strengthening health systems using innovative digital health technologies in Africa. Front Digit Health. 2022;4:854339. 35434700 10.3389/fdgth.2022.854339 PMC9008130

[10] Ojo A, Tolentino H, Yoon SS. Strengthening eHealth systems to support universal health coverage in sub-Saharan Africa. Online J Public Health Inform. 2021;13(3):E17. 10.5210/ojphi.v13i3.11550 35079321 PMC8769196

[11] Tilahun B, Teklu A, Mancuso A, Endehabtu BF, Gashu KD, Mekonnen ZA. Using health data for decision-making at each level of the health system to achieve universal health coverage in Ethiopia: The case of an immunization programme in a low-resource setting. Health Res Policy Syst. 2021;19(Suppl 2):48. 10.1186/s12961-021-00694-1 34380496 PMC8356368

[12] LaVenture M, et al. Developing an Informatics-Savvy Health Department: From discrete projects to a coordinating program. Part I: Assessment and Governance. J Public Health Manage Pract. 2017;23(3):325–327. 10.1097/PHH.0000000000000551 28350628

[13] Nyangena J, Rajgopal R, Ombech EA, et al. Maturity assessment of Kenya’s health information system interoperability readiness. BMJ Health Care Inform. 2021;28(1):e100241. 10.1136/bmjhci-2020-100241 PMC8252685 34210718

[14] Kipruto H, Muneene D, Droti B, et al. Use of digital health interventions in sub-Saharan Africa for health systems strengthening over the last 10 years: A scoping review protocol. Front Digit Health. 2022;4:874251. 10.3389/fdgth.2022.874251 35601887 PMC9120370

[15] Marongwe P, Chiboma I, Chitambala C, et al. Advancing National Health Information Systems Maturity: Lessons learned on implementing the Informatics-Savvy Health Organization (Isho) Assessment and Action Planning Framework for Health Leaders in Zambia [homepage on the Internet]. Rochester, NY; 2024 [cited 2024 Aug 16]. Available from: https://papers.ssrn.com/abstract=4771863

[16] World Health Organization. Monitoring the building blocks of health systems: A handbook of indicators and their measurement strategies [homepage on the Internet]. Geneva: World Health Organization; 2010 [cited 2024 Feb 15]. Available from: https://iris.who.int/handle/10665/258734

[17] Naeem M, Ozuem W, Howell K, Ranfagni S. A step-by-step process of thematic analysis to develop a conceptual model in qualitative research. Int J Qual Methods. 2023;22:16094069231205789. 10.1177/16094069231205789

[18] Vaismoradi M, Turunen H, Bondas T. Content analysis and thematic analysis: Implications for conducting a qualitative descriptive study. Nurs Health Sci. 2013;15(3):398–405. 10.1111/nhs.12048 23480423

[19] Ministry of Public Health of the DRC. National Development Plan Health Informatics 2020–2024. National Agency for Clinical Engineering, Information and Health Informatics (ANICiiS) (unpublished).

[20] Democratic Republic of The Congo Ministry of Public Health, Hygiene and Prevention. Quantified roadmap of health investments digital. (unpublished).

[21] Republic of Kenya. The Kenya Ministry of Health Virtual Academy. [cited 2024 Feb 15]. Available from: https://elearning.health.go.ke/

[22] Lal A, Ashworth HC, Dada S, Hoemeke L, Tambo E. Optimizing pandemic preparedness and response through health information systems: Lessons learned from Ebola to COVID-19. Disaster Med Public Health Prep. 2022;16(1):333–340. 10.1017/dmp.2020.361 33004102 PMC7642496

[23] Sherr K, Fernandes Q, Kanté AM, et al. Measuring health systems strength and its impact: experiences from the African Health Initiative. BMC Health Serv Res. 2017;17(Suppl 3):827. 10.1186/s12913-017-2658-5 29297341 PMC5763472

[24] Fritz J, Herrick T, Gilbert SS. Estimation of health impact from digitalizing last-mile Logistics Management Information Systems (LMIS) in Ethiopia, Tanzania, and Mozambique: A Lives Saved Tool (LiST) model analysis. PLoS One. 2021;16(10):e0258354. 10.1371/journal.pone.0258354 34695158 PMC8544866

[25] Biru A, Birhan D, Melkamu G, Gebeyehu A, Omer AM. Pathways to improve health information systems in Ethiopia: current maturity status and implications. Health Res Policy Syst. 2022;20(1):78. 35768819 10.1186/s12961-022-00860-z PMC9245200

[26] Kolukısa Tarhan A, Garousi V, Turetken O, Söylemez M, Garossi S. Maturity assessment and maturity models in health care: A multivocal literature review. Digit Health. 2020;6:2055207620914772. 10.1177/2055207620914772 32426151 PMC7216018

[27] Iyer HS, Chukwuma A, Mugunga JC, Manzi A, Ndayizigiye M, Anand S. A comparison of health achievements in Rwanda and Burundi. Health Hum Rights. 2018;20(1):199–211. 30008563 PMC6039746

[28] Koumamba AP, Bisvigou UJ, Ngoungou EB, Diallo G. Health information systems in developing countries: Case of African countries. BMC Med Inform Decis Mak. 2021;21(1):232. 10.1186/s12911-021-01597-5 34348718 PMC8336100

[29] Building a strong and interoperable digital health information system for Uganda – MEASURE evaluation [homepage on the Internet]. [cited 2024 Feb 15]. Available from: https://www.measureevaluation.org/resources/publications/fs-18-296.html

[30] Ndoungue VF, Tiwoda C, Gnigninanjouena O, Bataliack S, Mbondji E, Labat A. National Health Observatory: A tool to strengthen the health information system for evidence-based decision making and health policy formulation in Cameroon. Health Policy OPEN. 2022;3:100085. 10.1016/j.hpopen.2022.100085 36523955 PMC9742855

[31] Rumunu J, Wamala JF, Konga SB, et al. Integrated disease surveillance and response in humanitarian context: South Sudan experience. Pan Afr Med J. 2022;42(Suppl 1):13. 10.11604/pamj.supp.2022.42.1.33779 PMC9474851 36158932

[32] Woods L, Dendere R, Eden R, et al. Perceived impact of digital health maturity on patient experience, population health, health care costs, and provider experience: Mixed Methods Case Study J Med Internet Res 2023;25:e45868. 37463008 10.2196/45868 PMC10394505

[33] Snowdon A, Hussein A, Olubisi A, Wright A. Digital maturity as a strategy for advancing patient experience in US hospitals. J Patient Exp. 2024;11:23743735241228931. 10.1177/23743735241228931 38361832 PMC10868476

[34] Liang Z, Xu M, Liu G, et al. Patient-centred care and patient autonomy: Doctors’ views in Chinese hospitals. BMC Med Ethics. 2022;23:38. 10.1186/s12910-022-00777-w 35395761 PMC8994393

[35] Lane J, Andrews G, Orange E, et al. Strengthening health policy development and management systems in low- and middle- income countries: South Africa’s approach. Health Policy OPEN. 2020;1:100010. 10.1016/j.hpopen.2020.100010 37383321 PMC10297791

